# Invariant NKT Cells as Novel Targets for Immunotherapy in Solid Tumors

**DOI:** 10.1155/2012/720803

**Published:** 2012-10-17

**Authors:** Karsten A. Pilones, Joseph Aryankalayil, Sandra Demaria

**Affiliations:** Department of Pathology, NYU School of Medicine, 550 First Avenue, MSB-521, New York, NY 10016, USA

## Abstract

Natural killer T (NKT) cells are a small population of lymphocytes that possess characteristics of both innate and adaptive immune cells. They are uniquely poised to respond rapidly to infection and inflammation and produce cytokines that critically shape the ensuing adaptive cellular response. Therefore, they represent promising therapeutic targets. In cancer, NKT cells are attributed a role in immunosurveillance. NKT cells also act as potent activators of antitumor immunity when stimulated with a synthetic agonist in experimental models. However, in some settings, NKT cells seem to act as suppressors and regulators of antitumor immunity. Here we briefly review current data supporting these paradoxical roles of NKT cells and their regulation. Increased understanding of the signals that determine the function of NKT cells in cancer will be essential to improve current strategies for NKT-cell-based immunotherapeutic approaches.

## 1. Introduction

Natural killer T cells constitute a small population of lymphocytes defined by the expression of both *αβ* T-cell receptors (TCR) and lineage markers of natural killer (NK) cells. NKT cells possess unique phenotypic and functional characteristics that set them apart from conventional T cells. The TCR repertoire expressed by a major subset of NKT cells is highly invariant—a canonical *α*-chain (V*α*24-J*α*18 in humans; V*α*14-J*α*18 in mice) associated with a limited spectrum of *β* chains (V*β*11 in humans; V*β*8.2, V*β*2, V*β*7 in mice), in stark contrast to the highly polymorphic TCRs expressed by *αβ* T-cells [1, 2]. Such NKT cells are referred to as type I or invariant NKT (iNKT) cells. On the other hand, another population of NKT cells called nonclassical or noninvariant type II NKT cells displays a more heterogenous *αβ* usage [3, 4]. Some studies suggest that type II NKT cells play an antagonistic role to type I NKT cells and generally have a regulatory role under conditions of immune dysregulation such as cancer [5, 6]. However, the lack of reagents to reliably identify this subset precludes the unequivocal demonstration of their function. This paper will focus exclusively on type I invariant NKT cells in solid tumors.

## 2. Characteristics of iNKT Cells

Unlike conventional *αβ* T cells that recognize peptide antigens presented by major histocompatibility (MHC) class I and II molecules, iNKT cells exclusively recognize glycolipid antigens presented on CD1d molecules [7]. MHC-like CD1d molecules are constitutively expressed by antigen-presenting cells (APCs) such as dendritic cells (DCs), B cells, and macrophages capable of internalizing and processing lipid antigens prior to presentation on their surfaces [8]. CD1d molecules are also highly expressed in thymic stroma where they are required for development of iNKT cells [9].

Identifying the exact nature of the lipid antigens recognized by iNKT cells remains a major challenge. To date, the most well-characterized glycolipid ligand recognized by iNKT cells is *α*-galactosylceramide (*α*-GalCer) discovered initially in marine sponges (*Agelas*) from a screen of natural products with anticancer properties [10]. *α*-GalCer shows a strong affinity for CD1d molecules in both humans and mice. Recognition of CD1d-bound *α*-GalCer elicits a strong cytokine response by iNKT cells [11]. Due to this strong agonist activity *α*-GalCer has been used extensively to study the function of iNKT cells. However, since mammalian cells are incapable of synthesizing alpha-linked glycolipids, intense efforts are ongoing to identify natural ligands in humans and mice. Endogenous glycolipids such as glycophosphatidylinositol (GPI), ganglioside (GD3), and glycosphingolipid have been shown to activate iNKT cells but the physiological role for these ligands remains unclear [12–15]. The biological relevance of a putative endogenous glycolipid, isoglobotrihexosylceramide (iGB3), for human and mouse iNKT cells [12] has been questioned given the findings that mice deficient in iGB3 synthase develop a normal iNKT cell repertoire [16]. In 2008, Dhodapkar's group reported that plasma-derived lysophosphatidylcholine (LPC) from myeloma patients could stimulate interleukin (IL)-13 cytokine production in a small subset of CD1d-restricted T cells [17]. This was independently confirmed by the Gumperz lab that showed robust antigenicity of LPC in human iNKT cell clones [18]. Recently, the naturally occurring glycosphingolipid *β*-D-glucopyranosylceramide (*β*-GlcCer) has been found to potently stimulate iNKT cells. Its accumulation in LPS-stimulated bone-marrow-derived DC is thought to provide the self-antigen to initiate iNKT cell activation [19]. Interestingly, CD1d-dependent cross-presentation by DC of GD3 derived from human melanoma cells was shown to activate cytokine responses in iNKT cells, suggesting mechanisms whereby CD1d-negative tumors may influence the antitumor immune response [20]. Among endogenous CD1d ligands, the glycosphingolipid sulfatide and its isoforms are highly expressed in many organs and represent a major component of myelin sheath and pancreatic beta-cells [21, 22]. Sulfatides are recognized by type II NKT cells and have been implicated in the regulation of autoimmunity in multiple sclerosis and diabetes [23].

CD1d ligands with agonistic function for iNKT cells have also been identified in pathogens. Bacterial glycolipids, that is, phosphatidylinositol mannoside (PIM4), *α*-galacturonosylceramide (*α*-GalA-Cer), and *α*-glucuronosylceramide (*α*-GlcA-Cer), have been shown to stimulate cytokine production by iNKT cells [24–28]. These bacterial-derived glycolipids explain the critical dual role of iNKT cells in initiating rapid antibacterial immune responses characteristic of innate cells while also possessing antigen specificity [24–27].

Activation of iNKT cells is accompanied by the rapid and robust production of both T-helper 1 (Th1) and T-helper 2 (Th2) cytokines [29–31]. This requires engagement of costimulatory molecules such as CD40:CD40L and B7:CD28 pathways similar to conventional T cells [10, 32–34]. Both human and mouse iNKT cells also utilize, and are functionally constrained by, the same checkpoint receptors cytotoxic T-lymphocyte antigen 4 (CTLA-4) and programmed cell death-1 (PD-1) that regulate conventional T cells [35, 36].

Overall, iNKT cells are uniquely positioned to shape adaptive immune responses and have been demonstrated to play a modulatory role in a wide variety of diseases such as autoimmunity, infection, and cancer [37–39].

## 3. iNKT Cells as Effectors of Tumor Immunity

Initial evidence supporting an important role for iNKT cells in tumor immunity was drawn from studies using J*α*18 gene-targeted knockout mice that exclusively lacked iNKT cells [40]. iNKT-deficient mice exhibited significantly increased susceptibility to methylcholanthrene- (MCA-) induced sarcomas and B16F10 melanoma tumors [40], an effect reversed by the administration of liver-derived iNKT cells during the early stages of tumor growth [41]. IFN-*γ* production by iNKT cells, as well as NK cells and CD8+ effectors, was subsequently shown to be absolutely critical in tumor rejection [42].

**Figure 1:**
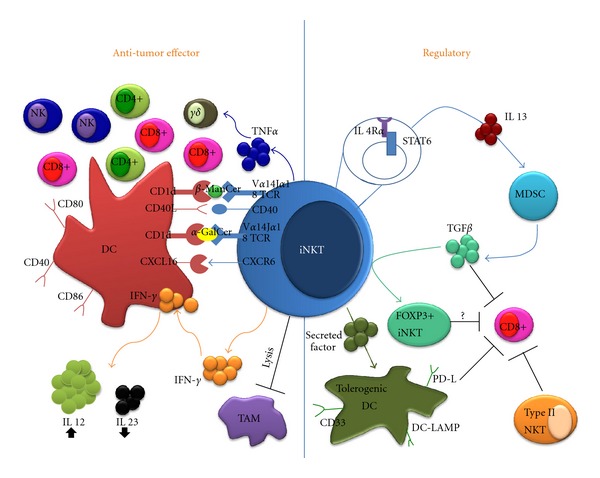
In cancer, iNKT cells play a dual role that can promote (left) or suppress (right) the antitumor immune response. In the presence of a strong activator (*α*-GalCer), iNKT cells promote the ability of DCs to prime effector cells through IL-12 production and upregulation of costimulatory molecules. Ligation of CD40L on the surface of DCs provides positive feedback enhancing iNKT cell activation. These events ultimately lead to downstream activation of antitumor effectors such as NK cells, CD8+, and CD4+ T-cells. Other iNKT agonists (*β*-ManCer) stimulate TNF-*α* production, leading to activation of antitumor *γδ* T-cells. iNKT cells may also promote antitumor immunity by directly killing protumorigenic macrophages (TAMs). On the other hand, IL-13 production by iNKT cells can trigger TGF-*β* production by suppressive MDSCs. TGF-*β* directly inhibits effector CD8+ activity and can induce FoxP3 expression in iNKT cells. iNKT cells can also induce DCs to acquire a tolerogenic phenotype, including expression of DC-LAMP, PD-L, and CD33. Data suggest that type II NKT cells perform always immunosuppressive functions in cancer.

Most of the evidence supporting an antitumor function for iNKT cells is derived from studies in mice demonstrating the ability of *α*-GalCer to inhibit tumor metastases [43] or suppress tumor development in several models, including sarcomas [44] and carcinomas [45, 46]. Although iNKT cells can directly recognize and kill CD1d-expressing malignant cells [47, 48], most solid tumors downregulate or do not express CD1d and thus remain immunologically invisible to iNKT-mediated cytotoxicity. The primary contribution of iNKT cells to tumor immunosurveillance occurs indirectly via the activation of iNKT cells by DC presenting *α*-GalCer. Activated iNKT cells then initiate a series of cytokine cascades that help boost the priming phase of the antitumor immune response [37]. The underlying mechanism is well characterized and involves enhancing IL-12p70 while inhibiting IL-23 cytokine production in DCs [49]. IFN-*γ* produced by *α*-GalCer-activated iNKT cells is widely believed to provide the initiating signal that skews the IL-12p70/IL-23 balance [49, 50]. The activation of iNKT cells also induces the upregulation of costimulatory molecules in DCs such as CD40, CD80, and CD86 [51]. Activated DCs reciprocally enhance expression of CD40L in iNKT cells, providing a positive feedback signal that amplifies the IFN-*γ* response [52]. Ligation of chemokine receptor CXCR6 on the surface of iNKT cells by its ligand CXCL16 expressed on APCs can also provide costimulatory signal resulting in robust *α*-GalCer-induced iNKT activation [53, 54]. These events ultimately lead to downstream activation of critical effectors of antitumor immunity including NK cells, cytotoxic CD8+ cells, and helper CD4+ cells (Figure 1) [51, 55].

The key role played by IFN-*γ* in the iNKT-mediated antitumor response was demonstrated in studies showing abrogation of the antitumor response induced by *α*-GalCer in IFN-*γ* ^−/−^ mice [56, 57]. Interestingly, the antitumor activity induced by a recently discovered iNKT agonist, *β*-mannosyl-ceramide (*β*-ManCer), in mice bearing the CT26 colon carcinoma or B16F10 melanomas was mediated primarily by nitric oxide species (NOS) and tumor necrosis factor alpha (TNF-*α*) [58]. Inhibition of either NOS by N-nitro-L-arginine-methyl ester (L-NAME) or inactivation of TNF-*α* by repeated injections of etanercept (TNF-*α*R-Fc) completely abrogated the antitumor effect of *β*-ManCer but not *α*-GalCer-treated tumor-bearing mice. Similarly, Van der Vliet and coworkers have demonstrated that activated iNKT cells produce significant levels of TNF-*α* that potentiate the activation of a subset of *γδ* T cells (V*γ*9V*δ*2) with effector activity against solid tumors [59].

Consistent with the ability of iNKT cells to modulate downstream effectors, Park and coworkers showed that NKT cells play a critical role not only in the generation of effectors during the priming phase but also in the maintenance and augmentation of secondary antitumor immune responses [60]. Effector CD8+ T cells were obtained from mice immunized with *α*-GalCer-loaded tumor extracts and adoptively transferred into recipient CD1d^+/−^ mice. The proliferation of the adoptively transferred cells correlated with the ability to reject a tumor challenge. No protection was seen in CD1d^−/−^ recipients that lacked all NKT cells or in J*α*18^−/−^ recipients that lack only iNKT cells, implicating iNKT cells in mediating protective antitumor responses [60]. Similarly, iNKT cells were shown to support secondary antitumor responses by adoptively transferred CD4+ T cells [61].

Intriguingly, iNKT cells have also been shown to specifically target the killing of CD1d-positive tumor-associated macrophages (TAMs), a highly plastic subset of inflammatory cells derived from circulating monocytes that perform immunosuppressive functions [62]. TAMs are known to be the major producers of IL-6 that promotes proliferation of many solid tumors, including neuroblastomas and breast and prostate carcinomas [63, 64]. Direct CD1d-dependent cytotoxic activity of iNKT cells against TAMs suggests the importance of an alternative indirect pathway by which iNKT cells can mediate antitumor immunity, especially against solid tumors that do not express CD1d.

## 4. Regulatory Functions of iNKT Cells

 Accumulated data from murine autoimmune disease models have provided compelling evidence that iNKT cells can also exert regulatory effects on inflammatory immune responses. In mouse models of type I diabetes [65, 66], rheumatoid arthritis [67, 68], and experimental autoimmune encephalitis (EAE) [69–71], iNKT cells played key roles in establishing tolerance and preventing autoimmune pathology. Likewise, iNKT cells have been attributed a suppressor role in cell-mediated antitumor immunity in some mouse tumor models [72, 73]. Interestingly, in patients with primary hepatocellular or metastatic cancer, one study found that CD4+ iNKT cells that produced high levels of Th2 cytokines and had low cytolytic activity were enriched within the tumor, suggesting that these cells may contribute to generate an immunosuppressive microenvironment [74].

Data suggesting that polarization towards Th2 cytokines secretion is an important mechanism of immunoregulation by iNKT cells are derived from studies that investigated the protective effects of *α*-GalCer in autoimmune diseases. In a model of experimental autoimmune encephalomyelitis, tolerance induction by *α*-GalCer required both IL-4 and IL-10 [70]. Singh and coworkers showed that production of IL-4 by iNKT cells was crucial while IL-10 was dispensable for disease protection in a mouse model of type 1 diabetes [75].

In initial studies using a mouse model of transformed recurrent fibrosarcoma, suppression of antitumor CD8+ cytolytic T cells was mediated primarily by NKT cells that produced IL-13 through an IL-4R*α*-STAT6-dependent pathway [72]. IL-13 was subsequently shown to stimulate myeloid-derived suppressor cells (MDSCs) to produce transforming growth factor (TGF)-*β*, a pleiotropic cytokine with powerful immunosuppressive functions [76]. On the other hand, other immunoregulatory pathways independent of the IL-4/STAT6/TGF-*β* axis could be exploited by NKT cells in other solid tumors to downregulate antitumor response [77]. To resolve this paradoxical dual pro- and antitumor function of NKT cells, Terabe and coworkers proposed that type II noninvariant NKT cells were responsible for the regulatory role while iNKT cells were responsible for promoting tumor rejection. This concept was based on experiments that compared the antitumor response in CD1d^−/−^ mice, which lack all NKT cells, and J*α*18^−/−^ mice, which lack only iNKT cells. In wild-type mice, the implantation of the 15-12RM fibrosarcoma typically results in initial growth followed by a period of spontaneous regression but tumors subsequently recur [72]. This tumor growth pattern was recapitulated in J*α*18^−/−^ mice but in CD1d^−/−^ mice tumors were unable to regrow, implicating type II NKT cells in suppressing the spontaneous antitumor response [78]. Based on a similar comparison of CD1d^−/−^ and J*α*18^−/−^ mice, type II NKT cells were also implicated in the suppression of antitumor response responsible for controlling the growth of implanted CT26 colon carcinoma and inhibiting the development of pulmonary metastases following intravenous injection of tumor cells [78–80]. Furthermore, selective stimulation of type II NKT cells by sulfatide was sufficient to override the protective effects of *α*-GalCer-stimulated type I NKT cells in the 15-12RM model [5]. Based on these collective data, Terabe and coworkers proposed a functional dichotomy between the two major subsets of NKT cells in which the iNKT (type I) cells activate antitumor responses while type II cells negatively regulate them. However, definitive conclusions on the regulatory nature of type II NKT cells can only be derived from tumor studies in knockout mice specific for type II NKT cells. The absence of such strain, the lack of reliable reagents that exclusively identify and stimulate type II NKT cells, and contradictory data from autoimmune disease models showing induction of peripheral tolerance from *α*-GalCer-activated iNKT cells continue to challenge the paradigm of a strict functional compartmentalization of NKT subsets.

Studies done in other tumor models strongly suggest that more complex mechanisms may be at play in the downregulation of immunity by iNKT cells, which cannot be fully explained by subset compartmentalization. For example, the poorly immunogenic and spontaneously metastatic 4T1 carcinoma showed comparable growth of the primary tumor in syngeneic wild-type, CD1d^−/−^, and J*α*18^−/−^ mice [78, 81]. However, in J*α*18^−/−^ mice there were significantly fewer lung metastases [81] and improved survival after surgical removal of the primary tumor [78], suggesting that in the absence of iNKT cells mice develop a spontaneous effector response, a conclusion supported by CD8 depletion experiments [81]. In addition, we showed that the lack of iNKT cells in J*α*18^−/−^ mice significantly enhanced the antitumor CD8+ response elicited by treatment with local radiation and CTLA-4 checkpoint blockade, further supporting a regulatory role of iNKT cells [81]. On the other hand, Terabe and co-workers reported that, while J*α*18^−/−^ mice showed better tumor control than wild-type mice, almost all of tumor-bearing CD1d^−/−^ mice survived beyond 80 days [78]. This is especially impressive in the 4T1 model since the poorly immunogenic and highly invasive tumors are particularly hard to cure and, in the absence of any intervention, mice rarely survive beyond 40 days [82, 83]. They subsequently concluded from the 4T1 data that only type II NKT cells were critical for immunosuppression while type I ones were dispensable [78]. However, reinterpretation of this data may be necessary in light of the recent findings of CD1d transcripts in 4T1 tumors [81] that could potentially act as a rejection neoantigen in CD1d^−/−^ mice. Although surface CD1d expression of 4T1 tumor cells in vitro was negligible [78], in vivo tumors can express CD1d albeit at low levels (K. A. Pilones and S. Demaria unpublished data.)

 Compelling data from Jenny Gumperz's lab has indicated that the interaction between iNKT cells and CD1d-expressing APCs, such as DCs and macrophages, is a key determinant of later differentiation into suppressor APCs [84–87]. Soluble factors secreted by iNKT cells induced regulatory licensing during monocyte differentiation into myeloid DCs. Such tolerogenic DCs were able to suppress in vitro proliferation and IFN-*γ* production of stimulated peripheral blood mononuclear cells (PBMCs), supporting the hypothesis that iNKT cells suppress adaptive immunity via induction of tolerogenic DCs [86]. Phenotypically, iNKT-licensed DCs expressed conventional differentiation markers CD11c, CD11b, and HLA-DR but also expressed intracellular DC-LAMP (myeloid DC marker) and CD33 (also found in immunosuppressive MDSC). Upregulated expression of programmed cell death ligands (PD-Ls) on myeloid APCs hinted at possible shared mechanisms of tolerance induction by regulatory T cells (Tregs) and iNKT cells [84, 86]. Interestingly, iNKT cells have recently been shown to acquire FoxP3 expression following exposure to TGF-*β*, a process known to generate inducible Tregs [88].

## 5. Manipulating iNKT Cells for Cancer Treatment

Based on the initial successes in preclinical studies that demonstrate the potent antitumor activity of iNKT cells, intense efforts have been made in the last decade to initiate iNKT-based immunotherapeutic approaches for the treatment of cancer. Currently, these can be classified under three broad strategies that involve (a) direct injection of *α*-GalCer, (b) reinfusion of autologous DC loaded ex vivo with *α*-GalCer, or (c) autologous transplant of ex vivo expanded iNKT cells (summarized in Table 1).

Early clinical trials of direct *α*-GalCer injection in cancer patients were met with little success. In a phase I study in patients with solid tumors, the intravenous infusion of soluble *α*-GalCer was well tolerated over a wide range of doses; however, induction of cytokine secretion (TNF-*α* and GM-CSF) was seen only in patients with relatively high iNKT frequencies before treatment [89]. More importantly, none of the patients showed signs of any clinical improvement.

The idea that autologous dendritic cells preloaded with *α*-GalCer would make better iNKT stimulators was born out of animal studies showing a more robust activation of iNKT cells in vivo that resulted in improved tumor control [96, 97]. The feasibility of this approach has been studied in several phase I clinical trials employing *α*-GalCer-pulsed DCs delivered either intravenously or injected directly into the nasal submucosa [91–93]. Both routes were well tolerated by patients. However, no definitive conclusions could be derived on the extent of iNKT stimulation since iNKT numbers were highly variable between patients.

Several data indicate an impairment of iNKT cell number and function in cancer patients [98, 99]. Functional defects have been reported in malignant lymphoma patients whose iNKT cells failed to proliferate in response to ex vivo stimulation [100]. Furthermore, cytokine production by iNKT cells from patients with advanced prostate cancer were skewed towards Th2 cytokines production [99]. Development of protocols to expand iNKT cells from patients in vitro has allowed testing the notion that reconstitution of iNKT cells in cancer patients could be therapeutically beneficial [101, 102]. Indeed, the adoptive transfer of ex vivo expanded autologous iNKT cells was tolerated well in a small cohort of nonsmall cell lung cancer patients [90]. Although subsequent expansion of iNKT cells was observed in a few patients, none showed partial or complete remission [90].

## 6. Future Directions for iNKT-Based Cancer Immunotherapy (Figure 2)

### 6.1. Th1-Inducing iNKT Ligands

Since the discovery of *α*-GalCer as a potent activator of iNKT cells, several other synthetic CD1d-binding lipid antigens modified from *α*-GalCer have been identified. More importantly, these have been found to differentially activate cytokine response in iNKT cells. For instance, truncation of the acyl and sphingosine chain of *α*-GalCer has been shown to favor IL-4 production [71] while modification of a carbon glycoside analogue (*α*-C-GalCer) drove predominantly an IFN-*γ* response [103]. Therefore, it may be possible to fine-tune the function of iNKT cells to elicit predominantly Th1 or Th2 responses.

### 6.2. Combination Therapy

 Preclinical studies in mouse tumor models have provided the proof of principle that iNKT-based immunotherapy can be rationally combined with other treatments [104–106]. In two independent studies, the immunomodulatory property of a thalidomide derivative (lenalidomide) was demonstrated to enhance expansion and Th1 polarization of iNKT cells in healthy volunteers and in patients with multiple myeloma [107, 108]. These combinatorial strategies suggest that targeting multiple immune components is a promising approach to attain maximal antitumor effects.

### 6.3. More Efficient Licensing of DC

 Phase I studies demonstrate the feasibility of autologous adoptive transfer of *α*-GalCer-loaded DCs as a novel approach to augment iNKT numbers in some cancer patients (Table 1). Additional studies are needed to fine-tune this approach to further improve maturation and cross-priming ability of DCs that reliably and predictably result in sustained iNKT cell proliferation in vivo.

### 6.4. Strategies to Inhibit NKT Cells with Regulatory Function

 Data in mice suggest that, at least in some settings, iNKT cells perform regulatory functions and dampen the antitumor immune response induced by the combination of immunotherapy and local radiotherapy [81]. Although the mechanisms of this effect remain to be identified, some tumors have been shown to condition iNKT cells by releasing CD1d ligands that impair iNKT cell antitumor activity [109]. Strategies to block the regulatory functions of iNKT cells, or interfere with their tumor-mediated conditioning, for example by blocking CD1d, could be beneficial to improve the effects of immunotherapy and other treatments. 

### 6.5. CD1d-Expressing Tumor Cells as Vehicle

 DCs loaded with *α*-GalCer have proven to be more effective in eliciting sustained effector IFN-*γ* responses in iNKT cells without inducing anergy that usually follows the rapid cytokine response induced by free *α*-GalCer [96]. Thus far, several phase I clinical trials have demonstrated the feasibility and safety profile of this approach in mitigating iNKT antitumor responses (Table 1). Alternatively, iNKT cells are also able to recognize *α*-GalCer loaded on CD1d-expressing tumor cells and mount an antitumor response that inhibited experimental metastases and rejected subcutaneous challenge of tumor cells [110, 111]. Interestingly, iNKT activation by *α*-GalCer-loaded tumor cells obviated the need for additional costimulation [110]. Potentially, this approach could be clinically relevant especially for patients with hematologic cancers where tumor cells can easily be harvested as APCs. It will be necessary to screen tumors for CD1d expression which may require additional transfection before these can be loaded ex vivo and eventually reinfused back to the patient.

### 6.6. iNKT Cell Agonists as Vaccine Adjuvants

The adjuvant properties of *α*-GalCer have been exploited to enhance protective immunity elicited by vaccines directed largely against infectious diseases [112–115]. Proof-of-principle studies have further shown that the immunogenicity of a model vaccine antigen can be enhanced by *α*-GalCer and improve priming of antigen-specific T-cell responses capable of delivering protective antitumor immunity [51, 116]. Future applications of this technology will involve not only adjuvant studies with other vaccine delivery systems but also design of glycolipids with superior immune adjuvant effects. 

## 7. Conclusion

It is becoming increasingly clear that iNKT cells perform complex functions in cancer. Although functional heterogeneity of NKT cell subsets may explain some of their opposite roles in tumor immunity, additional studies are needed to clarify the nature of regulatory NKT cells. The tumor microenvironment is likely to play an important role in conditioning iNKT to acquire regulatory functions. There is relatively little information about the expression of glycolipids and other CD1d ligands by cancer cells. Characterization of the CD1d ligand repertoire of tumors may identify novel ligands with important effects on the antitumor immune response.

While results of clinical trials based on iNKT cell stimulation with *α*-GalCer have not matched the expectations, renewed efforts to understand the fine regulation of this critical T-cell subset are warranted. A thorough understanding of the balance between stimulatory and regulatory functions of iNKT cells is essential for the development of strategies that overcome regulatory mechanisms while promoting antitumor effects. iNKT-based immunotherapeutic approaches hold great potential for the treatment of cancer and promise to become an integral part of future immunotherapy strategies.

## Figures and Tables

**Figure 2:**
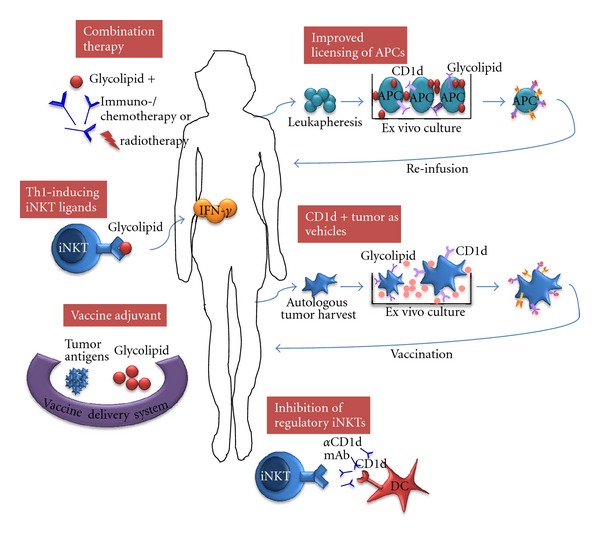
Multiple opportunities exist for innovations in iNKT-based cancer immunotherapy. Development of synthetic glycolipids that promote Th1 cytokine production by iNKT cells and the use of these agonists, including *α*-GalCer, as powerful adjuvants in cancer vaccines. Combinatorial approaches of iNKT stimulation with standard chemo-/radiotherapy or novel therapies that target other immune cells may result in synergistic effects. Blocking the activation of regulatory iNKT cells will be beneficial in tumors where regulatory iNKT cells play a key suppressor role. Optimization of protocols for ex vivo loading and maturation of autologous DCs will ensure consistent and reliable stimulation of iNKT cells in vivo. CD1d-expressing tumor cells can also be used as source of iNKT-stimulating APCs.

**Table 1:** Summary of NKT-based phase I/II clinical trials.

Tumor	Number of patients	Treatment regimen	Summary of study results	Reference
(A) Direct *α*-galactosylceramide injection				

Solid tumors	24	*α*-Galactosylceramide i.v. (40–4800 *μ*g/m^2^)	(1) No dose-limiting toxicity (2) Increase serum TNF-*α* and GM-CSF in 5 of 24 patients (3) Response dependent on pre-existing number of NKT cells which were significantly lower in cancer patients	Giaccone et al., 2002 [89]

(B) Infusion of ex vivo expanded NKT cells				

Nonsmall cell lung cancer	6	V*α*24NKT cells restimulated with *α*-galactosylceramide-pulsed PBMCs	(1) No adverse effects (2) Highest dose induced expansion of NKT cells (in 2 of 3 patients) and IFN-*γ* producing cells (in 3 of 3 patients)	Motohashi et al., 2006 [90]

(C) Ex vivo generated DC loaded with *α*-GalCer				

Nonsmall cell lung cancer	11	*α*-Galactosylceramide-pulsed dendritic cells	(1) No severe toxicities (2) Increase NKT cells in 1 of 11 patients (3) No partial or complete response seen	Ishikawa et al., 2005 [91]
Head and neck cancer	9	*α*-Galactosylceramide-pulsed APC	(1) No serious toxicities (2) Increase NKT cells in 4/9 patients (3) Increase NK activity in 8/9 patients	Uchida et al., 2008 [92]
Nonsmall cell lung cancer	23	*α*-Galactosylceramide-pulsed PBMC cultured with IL-2/GM-CSF	(1) No severe toxicities (2) Stable disease in 5 (3) Increased IFN response correlated with survival	Motohashi et al., 2009 [93]
Nonsmall cell lung cancer	4	*α*-Galactosylceramide-pulsed APC	(1) Increase in NKT cells in lung tumors (2) Highest IFN-*γ* production following in vitro restimulation of TILs with *α*-galactosylceramide	Nagato, K et al 2012 [94]

(D) Combination strategies				

Head and neck squamous cell cancer	8	In vitro expanded NKT + *α*-galactosylceramide-pulsed APC	(1) Transient and mild adverse effects (2) Increased NKT cells and IFN-*γ* secretion in 7 of 8 patients (3) Partial response in 3 patients, stable disease in 4, progressive disease in 1	Kunii, N et al, 2009 [95]
